# Supplementary material for: The Cdc25 and Ras1 Proteins of Candida albicans Influence Epithelial Toxicity in a Niche-Specific Way
Source: J Fungi (Basel). 2023 Feb 4;9(2):201. doi: 10.3390/jof9020201 (PMC9959987; doi:10.3390/jof9020201)
Supplement: Supplementary file 1 [file jof-09-00201-s001.zip › jof-2026613-supplementary.pdf]

Table S1 Statistical analysis of growth curve data

The area under the curve of each biological repeat during each condition was measured by making us of the R package Growthcurver [41]. The area under the curve of the different strains were compared and statistically analysed by making use of an ANOVA test with Bonferroni correction. A significant difference was found when $p \leq 0.05$*.

Table S1.1 Statistical comparison of growth curve data on 100 mM glucose

Strain comparison	Mean difference	Summary	Adjusted P value
WT vs. *cdc25*	10.42	*	0.0227
WT vs. *ras1*	18.89	***	0.0002
WT vs. *ras2*	8.496	Not significant	0.0715

Table S1.2 Statistical comparison of growth curve data on 100 mM fructose

Strain comparison	Mean difference	Summary	Adjusted P value
WT vs. *cdc25*	8.260	Not significant	0.2240
WT vs. *ras1*	14.77	*	0.0107
WT vs. *ras2*	4.025	Not significant	> 0.9999

Table S1.3 Statistical comparison of growth curve data on 100 mM galactose

Strain comparison	Mean difference	Summary	Adjusted P value
WT vs. *cdc25*	12.51	*	0.0266
WT vs. *ras1*	15.74	**	0.0053
WT vs. *ras2*	-2.329	Not significant	> 0.9999

Table S1.4 Statistical comparison of growth curve data on 5 mM glucose

Strain comparison	Mean difference	Summary	Adjusted P value
WT vs. *cdc25*	0.8189	Not significant	> 0.9999
WT vs. *ras1*	-1.180	Not significant	> 0.9999
WT vs. *ras2*	2.500	Not significant	> 0.9999

Table S1.5 Statistical comparison of growth curve data on RPMI

Strain comparison	Mean difference	Summary	Adjusted P value
WT vs. *cdc25*	-0.8357	Not significant	> 0.9999
WT vs. *ras1*	-1.163	Not significant	> 0.9999
WT vs. *ras2*	1.066	Not significant	> 0.9999

Figure S1 Filamentation experiment

In literature it was shown that a *cdc25* and *ras1* mutant had a defect in morphogenesis on serum containing medium, while a *ras2* mutant did not show abnormal filamentation [14, 16, 18]. As morphogenesis is important during different virulence traits, we confirmed if our strains show the same filamentation phenotype as in literature. Indeed, the phenotype of our mutant strains correlated with the findings in literature (Fig. S1).

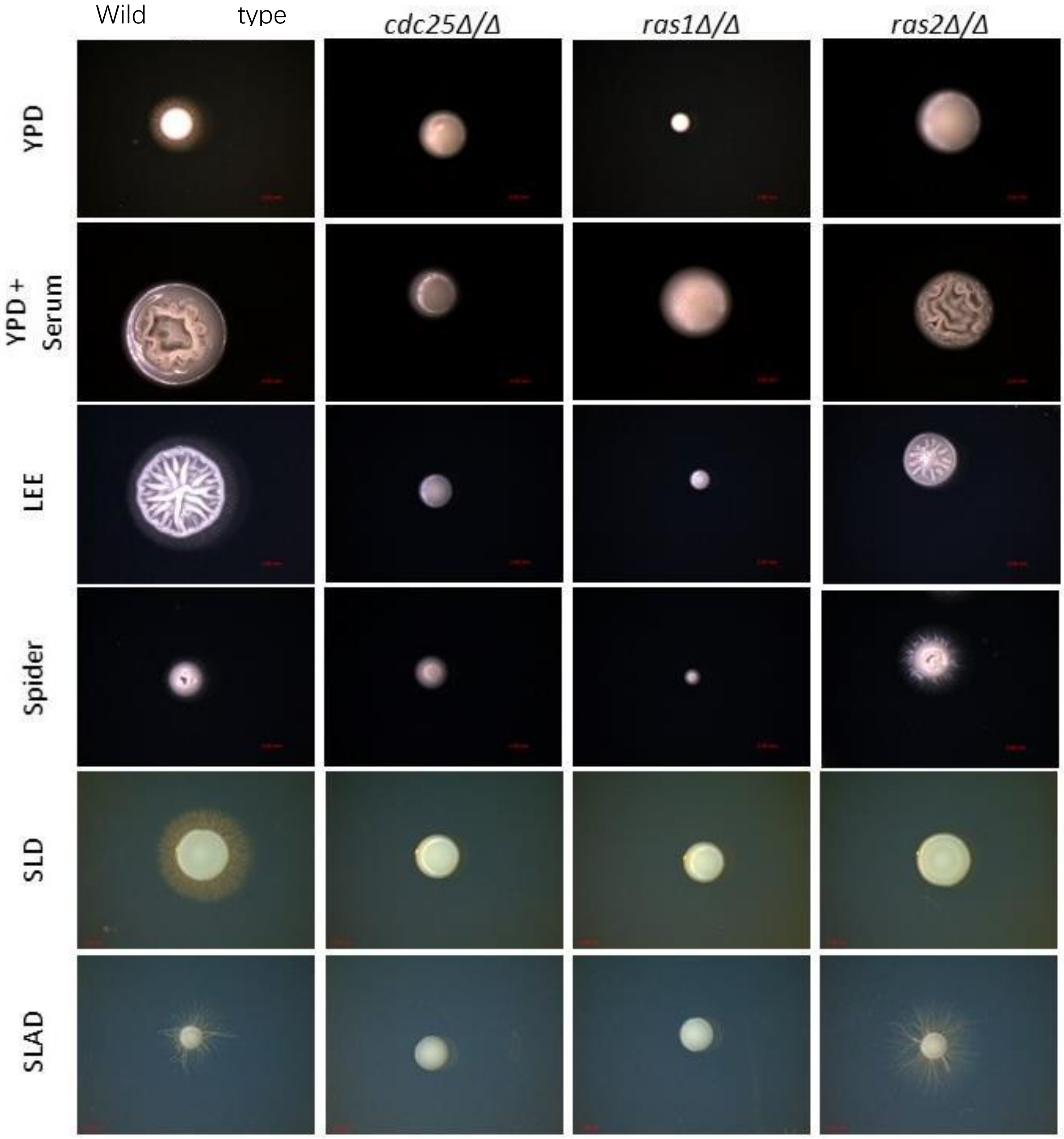

Figure S1: The *cdc25* and *ras1* mutant have a defective filamentation while the *ras2* mutant shows normal filamentation. The different strains were tested on different solid hyphal inducing media: YPD, YPD+serum, LEE, Spider, SLD and SLAD.
